# An immunotherapeutic approach to decipher the role of long non-coding RNAs in cancer progression, resistance and epigenetic regulation of immune cells

**DOI:** 10.1186/s13046-021-01997-5

**Published:** 2021-07-24

**Authors:** Krishnapriya M. Varier, Hemavathi Dhandapani, Wuling Liu, Jialei Song, Chunlin Wang, Anling Hu, Yaacov Ben-David, Xiangchun Shen, Yanmei Li, Babu Gajendran

**Affiliations:** 1 State Key Laboratory for Functions and Applications of Medicinal Plants, Guizhou Medical University, Guiyang, 550014 Guizhou Province People’s Republic of China; 2 The Key Laboratory of Chemistry for Natural Products of Guizhou Province and Chinese Academic of Sciences, Guiyang, 550014 Guizhou Province People’s Republic of China; 3 School of Pharmaceutical Sciences, Guizhou Medical University, Guiyang, 550025 Guizhou Province People’s Republic of China; 4 Department of Molecular Oncology, Cancer Institute (WIA), Chennai, 600020 India; 5 Department of Biosciences & Bioengineering, Indian Institute of Technology Bombay, Mumbai, 400076 India; 6 Guizhou University of Traditional Chinese Medicine, Guiyang, 550025 Guizhou Province People’s Republic of China

**Keywords:** LncRNAs, Adaptive and innate immune cells, Immunotherapy, Epigenetics, Autophagy

## Abstract

Immunotherapeutic treatments are gaining attention due to their effective anti-tumor response. Particularly, the revolution of immune checkpoint inhibitors (ICIs) produces promising outcomes for various cancer types. However, the usage of immunotherapy is limited due to its low response rate, suggesting that tumor cells escape the immune surveillance. Rapid advances in transcriptomic profiling have led to recognize immune-related long non-coding RNAs (LncRNAs), as regulators of immune cell-specific gene expression that mediates immune stimulatory as well as suppression of immune response, indicating LncRNAs as targets to improve the efficacy of immunotherapy against tumours. Moreover, the immune-related LncRNAs acting as epigenetic modifiers are also under deep investigation. Thus, herein, is a summarised knowledge of LncRNAs and their regulation in the adaptive and innate immune system, considering their importance in autophagy and predicting putative immunotherapeutic responses.

## Background

Cancer immunotherapy (CI) is a rapidly advancing treatment regimen supporting the primary mode of treatments like surgery, chemotherapy, radiation, and targeted therapy [1]. The conventional cancer treatment generally failed to produce beneficial responses in patients with late-stage disease treatment compared to early non-metastatic cancer patients. Besides, the traditional treatment modalities are often associated with side effects like disfigurement, cytotoxicity on normal cells, and hair loss [2]. However, CI has fewer side effects, and it aims to engage the immune system directly to recognize and eradicate tumor cells. The main attributes of CI are the specificity, breadth of response, and memory against the tumor antigens that can provide better clinical outcomes, and thus can increase the quality of life, particularly in metastatic patients [3].

Immunotherapy has a multi-faceted approach like the use of monoclonal antibodies, checkpoint inhibitors, cancer vaccine, and cell-based therapy [4]. These modalities can be either passive or active immune therapeutics. In passive immune therapeutics, the ICIs are variant monoclonal antibodies that block checkpoint receptors facilitating the T cell activation to clear the malignant cells. Recently employed ICIs are anti-PDL1s /anti-CTLA-4s that are passive immunotherapy agents [5]. While, active immune therapeutics specifically aim to generate durable, long-lasting memory responses, by utilizing immune cells like dendritic cells, which are the potent antigen-presenting cells and immunostimulatory cytokines [6]. However, there are several ongoing challenges for immunotherapy that make this passive and active immune therapeutics, highly challenging. One among them is that many persons fail to respond to the CI due to high mutation burdens [7]. This indicates that CI must be personalized through careful understanding and identification of the rate-limiting step working on a specific patient. These can be addressed by providing combinational strategies to overcome the cancer burden through utilizing high throughput technologies to characterize patients’ specific landscape like identifying a neoantigen and immune molecular signature pertain to the individuals, which may solve this to an extent [8]. Besides, several factors influence an effective CI like key evasion tactics including abrogated expression of cancer antigens and checkpoint receptor ligands which majorly prevent the entry of tumor-infiltrating lymphocytes (TILs) in tumor mass, decreased antigen presentation, b2-microglobulin alterations, severe exhaustion of T cells, and increased activation and recruitment of immunosuppressive cells or induction of suppressive cytokines such as IL-10 and TGF-β [9]. This concerns the specificity of CI in eliciting an immune response, overcoming the mechanisms that cancer cells employ to evade immune surveillance, and ensures that the activated immune cells have access to the malignant tissues. Hence, there is a persistent need in identifying more specific biomarkers, better predictive tools, and assays to identify patients who will respond to these immunotherapies.

To understand the depth of immune resistance, studies were focused on the tumor microenvironment, but they are unable to provide the detailed mechanisms of immune evasion to date. Intriguingly, recent studies showed that this issue can be resolved by considering the crucial involvement of non-coding RNAs in tumor cell-intrinsic factors that mediate tumor cell escape from immune surveillance [10, 11]. Through the evolution of high throughput sequence technology, rapid screening of non-coding RNAs was made possible in a limited time as never before [12, 13]. The research revealed that only a small portion of approximately 20,000 genes are protein-coding, which accounts for <2% of the human genome encodes, whereas 90% of the genome is transcribed into non-coding RNAs [14]. Of these non-coding RNAs, microRNAs (miRNAs) are widely studied and differentially expressed with several human pathologies, including cancer. Furthermore, miRNAs are the regulators of innate and adaptive immunity and their maintenance, helping in the generation of immune progenitors and their differentiation, modulating the functions of mature immune cells [15, 16].

Unlike miRNAs, long non-coding RNA is 200 nucleotides long, transcribed by RNA polymerase II, and regulated by splicing (via processing at the 5′ and 3′ ends), and exported to the cytoplasm [17]. LncRNAs respond to several cellular functions ranging from modification of chromatins leading to RNA stability through translational control. Biochemically, LncRNAs exert their function via RNA-RNA, RNA-DNA, or RNA-protein interactions [18, 19]. Moreover, LncRNAs are deregulated in several ailment conditions and could categorize cellular abnormalities leading to cancer. It may even account for delivering a prognostic value or therapeutic options for cancer patients [20, 21]. For example, MALAT1 is over-expressed in several cancer types, and knockdown of MALAT1 reduces both the proliferation and metastasis of tumor cells in several mouse models [22]. Besides, MALAT1 also acts as an epigenetic modifier by regulating the PRC2 complex [23]. Emerging studies also revealed the role of LncRNAs in differentiation, proliferation, and immune cells’ activation [24]. Thus, this review provides a critical analysis of LncRNAs in the regulation of cancer resistance by direct and indirect modification of T cells, B cells, Treg cells, natural killer cells (NKs), macrophages and myeloid-derived suppressor cells (MDSCs), and autophagosomes functionality, projecting it as a tool to treat various cancers, as a personalized treatment regimen.

## LncRNAs as modulators of the adaptive immune system

Adaptive immune responses are referred to the generation of antigen-specific lymphocytes by host organisms against the subsequent exposure of similar antigens or if reinfected with the same type of pathogen. The adaptive immune system consists of T cells and B cells [25].

### Inhibition of LncRNAs potentiates immunotherapeutic strategies of cytotoxic T lymphocytes (CTLs) by curtailing the functionality of T regulatory cells (Tregs)

The T lymphocytes stimulate the activation of the immune cell to fight against cancerous cells [26]. Moreover, T cells signal other immune cells to participate in the immune responses. There are several T cell sub-sets, which cause the death of cancer cells and prevent their progression. One among them is the CTLs which possess the surface expression of CD8^+^. These CTLs are involved in the destruction of targeted cells through apoptosis by utilizing the granules containing digestive enzymes or cytotoxic cytokines (Tumour Necrosis Factor-TNF and Interferon γ - IFN γ) [27]. Cancer cells are known to resists the effect of CTLs by reorganizing the cellular function through the expression of coding RNAs and LncRNAs leading to an altered cellular response to the native immune system. Recent studies also showed the role of LncRNAs in resisting the action of CTLs in cancers [28, 29].

**Fig. 1:**
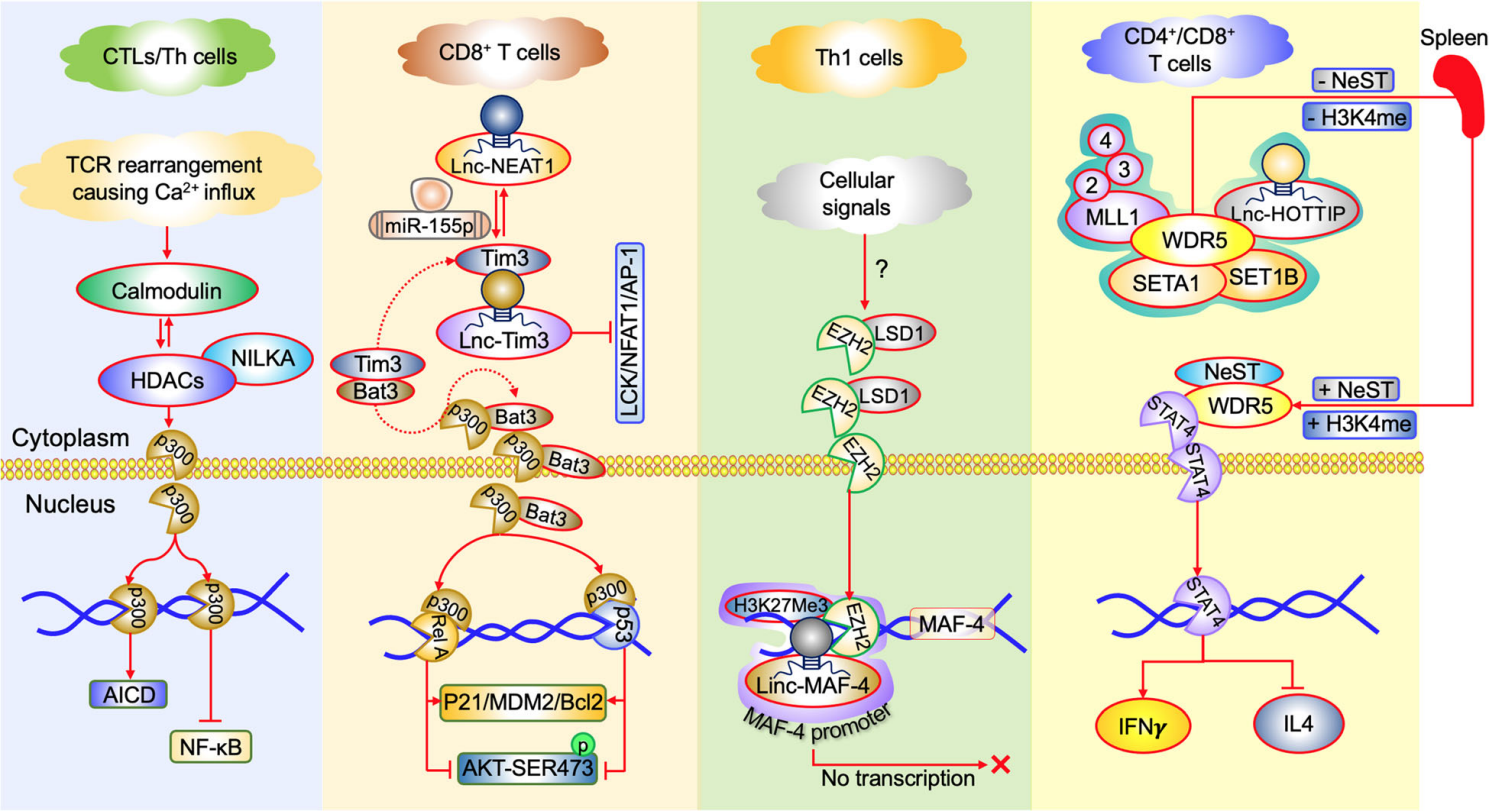
LncRNA mediated molecular pathways in T Cells. In CTLs and Th cells, due to TCR signalling, there may be a Ca^2+^ influx, which can activate calmodulin. This promotes deacetylation of NILKA promoter, causing nuclear translocation of p300 inhibiting NF-kB pathway and activating AICD. In CD8^+^ cells, Lnc-NEAT1 regulates miR-155p mediated Tim-3 activation. While binding of Lnc-Tim-3 to Tim-3 protein inhibit Lck/NFAT1/AP-1 pathway and permit the localisation of Bat-3 towards p300 mediated activation of RelA and p53. In Th1 cells, EZH2 binds to LSD1, causing nuclear localization of EZH2, which inactivates transcription of MAF-4 gene by recruiting Linc-MAF-4 and H3K27 trimethylation of the promotor. In CD4^+^/CD8^+^ cells, Lnc-NeST and Lnc-HOTTIP binds to WDR5 protein of SET/MLL/WRD5 complex. The NeST can even recruit STAT to WDR5 through methylation. STAT4, thus gets localised to nucleus causing activation of IFN gamma pathways

Nuclear-enriched autosomal transcript 1 (NEAT1) is a nuclear paraspeckle localized LncRNA reported in different types of cancer [30, 31]. In vivo experiments showed that the inhibition of NEAT1 decrease CD8^+^T cell apoptosis and gain active cytolytic function via the miR-155/Tim-3 pathway leading to enhanced immune activity (Fig. 1). Moreover, Tim-3 upregulated in exhausted T cells in tumors as well as in chronic infections. In turn, the increased expression of this Tim-3 leads to the death of CD8^+^ T cells. Hence Tim-3 has emerged as a potential immunotherapeutic target which is currently explored clinically [32]. However, Ji et al. (2018) [33] reported the role of Lnc-Tim-3 in hepatocellular carcinoma in the regulation of Tim-3 protein expressions. They stated that an increase of Lnc-Tim-3 specifically binds with Tim-3 protein and blocks its interaction with Bat-3 thus suppressing downstream Lck/NFAT1/AP-1 signaling, leading to nuclear localization of Bat-3, and enhancing p300-dependent p53 and RelA transcriptional activation of anti-apoptosis genes including MDM2 and Bcl2 leads to survival and proliferation of exhausted T cells (Fig. 1). This study indicates Lnc-TIM-3 can be used as to target to deploy the T cell exhaustion and proliferation.

Furthermore, Huang et al. (2018) [34] reported an increased expression of nuclear factor κB interacting long non coding RNA (LncRNA NKILA) in cytotoxic T lymphocytes leading to the poor prognosis in breast and lung cancer. But knockdown of NKILA significantly decreased the tumor growth by increasing the CTLs mediated activation induced cell death (AICD), by the histone-deacetylase (HDAC)-mediated P300 nuclear localization, post calcium influx and calmodulin activation (Fig. 1). Besides, long intergenic non-coding RNA (LincRNA) Tmevgpg1/ Nettoie Salmonella pas Theiler’s (NeST) identified in Thelier’s virus infection [35] revealed co-expression with interferon γ (IFNγ). It can stimulate epigenetic remodelling through its interaction with WDR5 mainly, depends on the transcription factors T-bet and STAT4. Hence this NeST is classified as an enhancer RNA [36, 37], was recognized for its proximity-based cis-transactivation of IFNγ. Moreover, the LincRNA-MAF-4 is also involved in the development of Thl response, mediated through the epigenetic silencing of the transcriptional factor MAF-4, by the recruitment of the chromatin modifiers like polycomb Group proteins (PRC1 and PRC2) which facilitates the deposition of H3K27me3 (transcriptional repression) by utilising the catalytic component partner mainly Enhance of Zeste Homolog 2 (EZH2). In addition, Lysine specific demethylase 1 (LSD1) also facilitates H3K4me3 (transcriptional activation) and H3K27me3 at their gene regulatory regions. Thus LincRNA-MAF-4 interacts with the transcriptional repressors LSD1 and EZH2 to facilitate the H3K27me3 trimethylation mark at the promoter of MAF to silence its expression (Fig. 1) [38, 39].

**Fig. 2:**
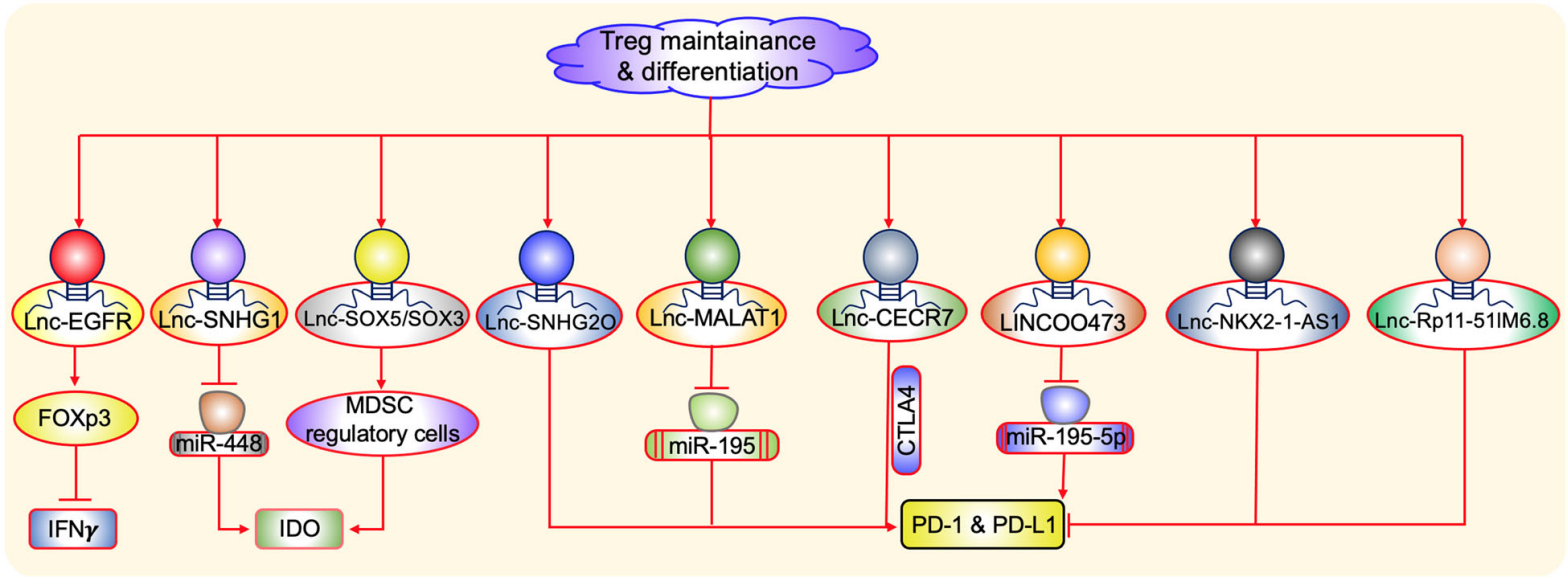
Molecular targets of LncRNAs responsible for T regulatory cells differentiation and inhibition of proliferation. In Treg cells, Lnc-EGFR stimulates FOXp3 causing inhibition of IFN. While Lnc-SNHG1 and Lnc-SOX5/SOX3 stimulates IDO production. Lnc-SNHG20, Lnc-MALAT1, Lnc-CECR7 and LINC00473 accelerates PD-1 and PD-L1. While Lnc-NKX2-1-AS1 and Lnc-Rp11-51IM6.8 inhibit PD-1 and PD-L1

Cancer cells mediated immune suppression by several mechanisms involves an accumulation of immune-suppressive tumor-infiltrating lymphocytes in the tumor microenvironment [40]. One among them is Tregs which are the most potent and well-studied suppressive phenotypes found in the tumor microenvironment. Increasing evidence shows that Treg cells may also play an important role in immune evasion mechanisms employed by cancer. In addition, recent evidence shows that tumor-derived LncRNA plays a pivotal role in Treg maintenance and differentiation [41]. For instance, Jiang et al. (2017) [42] pointed out the novel role of Lnc-epidermal growth factor receptor (EGFR) in differentiating Tregs, causing suppression of CTLs in hepatocellular carcinoma (Fig. 2). The study identified an upregulation of Lnc-EGFR in Tregs and was well-correlated with the expression of EGFR/Foxp3 and tumor size, but negatively correlated with IFN-γ expression. Even though many studies evidenced the FOXp3 Tregs are involved in the progression of the tumor, a recent study [43] showed the two different populations of Tregs called FOXp3 low and FOXp3 high have a different role in promoting tumor. In line with this Zemmour et al. (2017) [44] discovered the role of flicr (Foxp3 long intergenic noncoding RNA) in regulating the expression of FOXp3 in Treg. The increased expression of IL-2 followed by the T cell receptor (TCR) signaling are involved in intensifying the suppressive functions of Foxp3 in Tregs by activating conserved noncoding sequence-2 (CNS-2) enhancer by inhibiting flicr. Hence, there was an urge to study the role of LncRNA in controlling Treg functionality further to potentiate the immunotherapeutic strategies, including miR regulation, downstream of LncRNA regulatory mechanisms.

To understand the role of LncRNA and miR interplay in IDO production, Pei et al. (2018) [45] demonstrated the interaction of LncRNA-SNHG1 (Small nucleolar RNA host gene 1) with miR-448 in the regulation of the IDO expression in TILs. The study shows miR-448 negatively regulates the IDO expression, especially in CD4^+^ TILs not in CD4^+^ cells in circulating peripheral blood. Lnc-SNHG1 sponges miR-448 in turn, leading to upregulation of IDO expression in hepatocellular carcinomic cells [46] suggests LncRNA-SNHG1 might be used as potential therapeutic target in enhancing the immunotherapy by inhibiting the expression of LncRNA. Moreover, the increased expression of Lnc-SOX5 and SOX3, in the tongue and colon cancer was found to be inhibiting the T cell activity by upregulating IDO1 [47]. Previous studies also showed the IDO1 mediated immunosuppression facilitating the progression of tumor growth viz., upregulation of Treg and myeloid-derived suppressor cells (MDSC) regulatory cells (Fig. 2).

Besides, LncRNA is also involved in the regulation of CTLA4 and PD-L1 mediated Treg propagation. A recent report [48] showed the role of LncRNA RP11-571M6.8 driver LncRNA in promoting human cancers. The increased expression of LncRNA RP11-571M6.8 had lowered the expression of immune checkpoint proteins such as PD-1, PD-L1, and CTLA4. In addition, its expression is also well correlated with the exhausted Tregs, particularly in glioblastoma multiforme (GBM) datasets. Moreover, Zhou et al. (2019) [49] showed that LINC00473 LncRNA involved in the sponging of miR195-5p in pancreatic cancer leads to the upregulation of PD-L1 expression. In-line inhibition of LINC00473 causes enhancement of CTLs causing suppression of tumor progression. Moreover, Kathuria et al. (2018) [50] also reported that LncRNAs NKX2-1-AS1 and NXX2-1 in regulating the PD-L1 expression in lung cancer. Where NXX2-1 protein is involved in the upregulation of PD-L1 but the NKX2-1-AS1 LncRNA disturbs the upregulation of PD-L1 by reducing the CD274 gene transcript which codes for PD-L1 (Fig. 2). Thus suggest the screening the pattern of potential LncRNA which curtail the PD-L1 expression between cancer patients can predict the responders and non-responder in immune checkpoint therapy.

LncRNA small nucleolar RNA host gene 20 (SNHG20) also plays a crucial role in increasing the expression of PD-L1, p-ATM, and p-JAK1/2 in esophageal squamous cell carcinoma. The modulation of this signaling pathway promotes proliferation and metastasis of cancer cells [51]. LncRNA CECR7 (cat eye syndrome chromosome region, candidate 7) regulates the CTLA4 expression associated with the overall survival of hepatocellular carcinoma [52]. However, in diabetic pancreatic cancer, the expression of CECR7 was downregulated suggesting its expression pattern varies between different cancers (Fig. 2) [53].

However, MALAT1 was the most widely studied LncRNA which was initially identified in lung cancer [54]. Later it was also reported in driving H3K27me3 mediated repression of tumor suppressor genes through its interaction with the members of PRC2 complex mediating tumor progression and metastasis [55]. In addition, Zhao et al (2016) [56] revealed its role in regulating the innate immune response in NF-kB dependent manner. Moreover, a recent study by Wang et al. (2019) [57] indicated its involvement in sponging miR-195 causing upregulation of PD-L1, leading to immune escape and apoptosis of CTLs (Fig. 2). Hence, cancer specific expression of MALAT1 can be explored for targeted therapy as well as biomarkers in cancer.

### The role of Lnc RNA in B cell development and malignancy

**Fig. 3:**
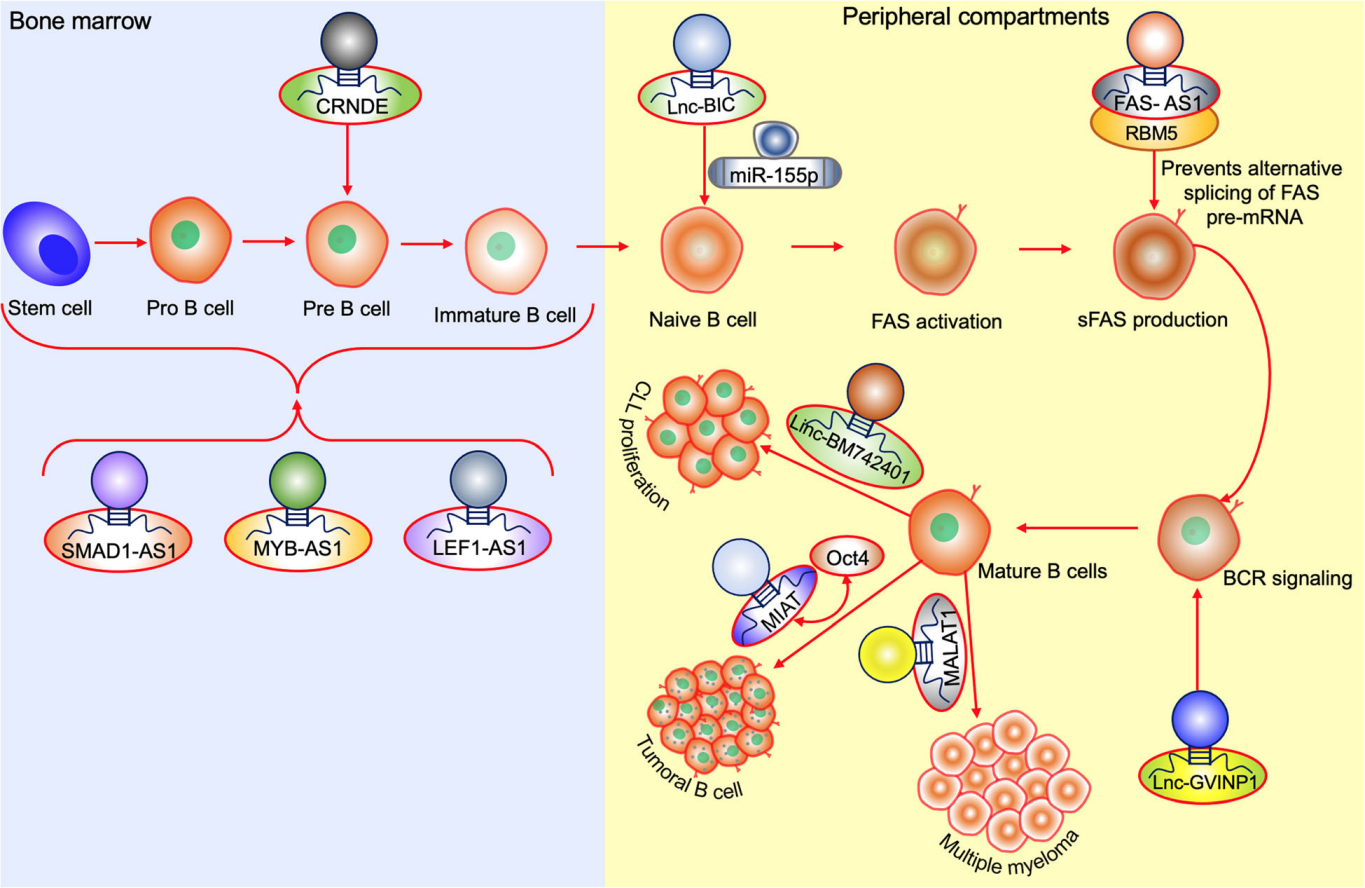
LncRNA mediated molecular pathways in B cell maturation. In bone marrow, the development of pro/pre/immature B cells are mediated by Lnc-CRNDE, SMAD1-AS1, MYB-AS1 and LEF1-AS1. lncRNA BIC plays a major role in hemotopoises peripheral compartments Naïve B cells and FAS activations are mediated by LncRNAs BIC and FAS-AS1. The BCR signalling is mediated by Lnc-GVINP1. Linc-BM742401, Oct-4 mediated MIAT and MALAT1 cause CLL proliferation, Tumoral B cell formation and multiple myeloma, respectively

The B cells play an important role as an initiator of humoral immune responses. In addition, B cells are also involved in the initiation of T cell immune responses, which are essential for regulating immune homeostasis [58]. Petri et al. (2015) [59] also showed the importance of LncRNA in the regulation of B cells in every stage of its differentiation. LncRNAs such as long intergenic ncRNAs (LincRNA) CTC 436K13.6 and SMAD1-AS1, MYB-AS1, and LEF1-AS1 which are antisense transcript encoded by their opposite sense strands possess a crucial function in the early B cell formations. Among the gene expressed, CRNDE (colorectal neoplasia differentially expressed) which found expressed in preB1, B2, and centroblasts (rapidly dividing B cells that achieve further diversification of the Ig repertoire through the somatic hypermutation of the V regions of Ig genes) and low in centrocytes (generate new antibody variants that are selected according to their affinity to the cognate antigen, ensuring increased affinity between the Ig and the antigen). Besides, the expression of this CRNDE function as the metabolic regulator in the early stage of B cell development is believed to favour the metabolic switch to aerobic glycolysis in tumor cells. Another LncRNA BIC (B-cell integration cluster) gives rise to miR-155-5p, and it plays a central role in hematopoiesis, inflammation, and immune response. The increased expression of BIC and miR-155 has been found in many mature B- cell malignancies [34]. Impaired production of soluble Fas receptor (sFAS) was associated with poor prognosis in non-Hodgkin’s lymphomas [60]. In line the expression of sFAS was tightly regulated by LncRNA FAS-AS1, where it binds with RNA binding motif protein5 (RBM5), preventing the alternative splicing of FAS pre-mRNA required for sFAS production. But the FAS-AS1 transcript was hyper methylated by over-expressed EZH2- enzymatic component of PRC2 complex leads to repression of FAS-AS1, in turn causes aberrant expression of sFAS in B-cell lymphoma. Parallelly, Zhou et al. (2016) [61] showed the downregulation of this LncRNA causes increased tumor size and lymph node metastasis in breast cancer patients. These data necessitate the use of EZH2 inhibitors in combination with the chemotherapeutic targets helps in augmenting the immune cell mediated cell killing. A recent bioinformatics analysis [62] in NSCLC revealed that LncRNA-GVINP1 was associated with regulation of the B cell receptor signaling pathway associated with the overall survival period of NSCLC patients. Kelin et al. (2010) [63] reported that LncRNA DLEU2 along with miR-15a/16-1, controls the proliferation of B cells, by the dysregulation of this cluster leading to uncontrolled proliferation in CLL (Fig. 3).

MIAT which was initially discovered in myocardial infarction [64] suggested constituting a regulatory loop with Oct4 in tumoral mature B-cell [65]. Further both MIAT and Oct4 were demonstrated to be essential for the tumour cell survival supporting the proliferation of malignant mature B cells [66]. Hence high expression of MIAT is associated to pathogenesis in cancer patients, can considered for potential biomarker as well as therapeutic target. LincRNA BM742401 was frequently methylated in chronic lymphocytic leukemia (CLL) [67] was previously reported that lower expression was correlated with poor survival in gastric cancer [68]. Particularly, the promoter region of BM742401 which was unmethylated in peripheral B-cells collected from healthy donors but methylated in 50% CLL samples at diagnosis. The methylation of this BM742401 in CLL samples was significantly associated with higher lymphocyte counts and correlated with the advanced stage. Further MALAT1 expression also significantly upregulated in multiple myeloma (MM) patients compared to treated or healthy donors. In addition, increased expression of MALAT1 was seen in relapsed MM patients [69]. These studies indicate the important role of non-coding RNA in curtailing the function of adaptive soldier’s which leads to tumour escape from immune surveillance causing tumour progression (Fig. 3).

## LncRNA act as regulators of innate immune warriors

The innate immune responses are not specific to a particular pathogen. They mainly depend on the pathogen-associated molecular patterns (PAMPs) and damage-associated molecular patterns (DAMPs) that are conserved features of pathogens [70]. The major effector cells of the innate immune system which are involved indirectly in targeting the cancer cells are natural killer cells (NKs), dendritic cells (DCs), macrophages, and myeloid-derived suppressor cells (MDSCs). In this section, we concentrated on the regulation of LncRNAs in NK, macrophages, and MDSCs.

### Reinforcing cancer resistance by curtailing the functionality of innate cytotoxic NK cells

**Fig. 4:**
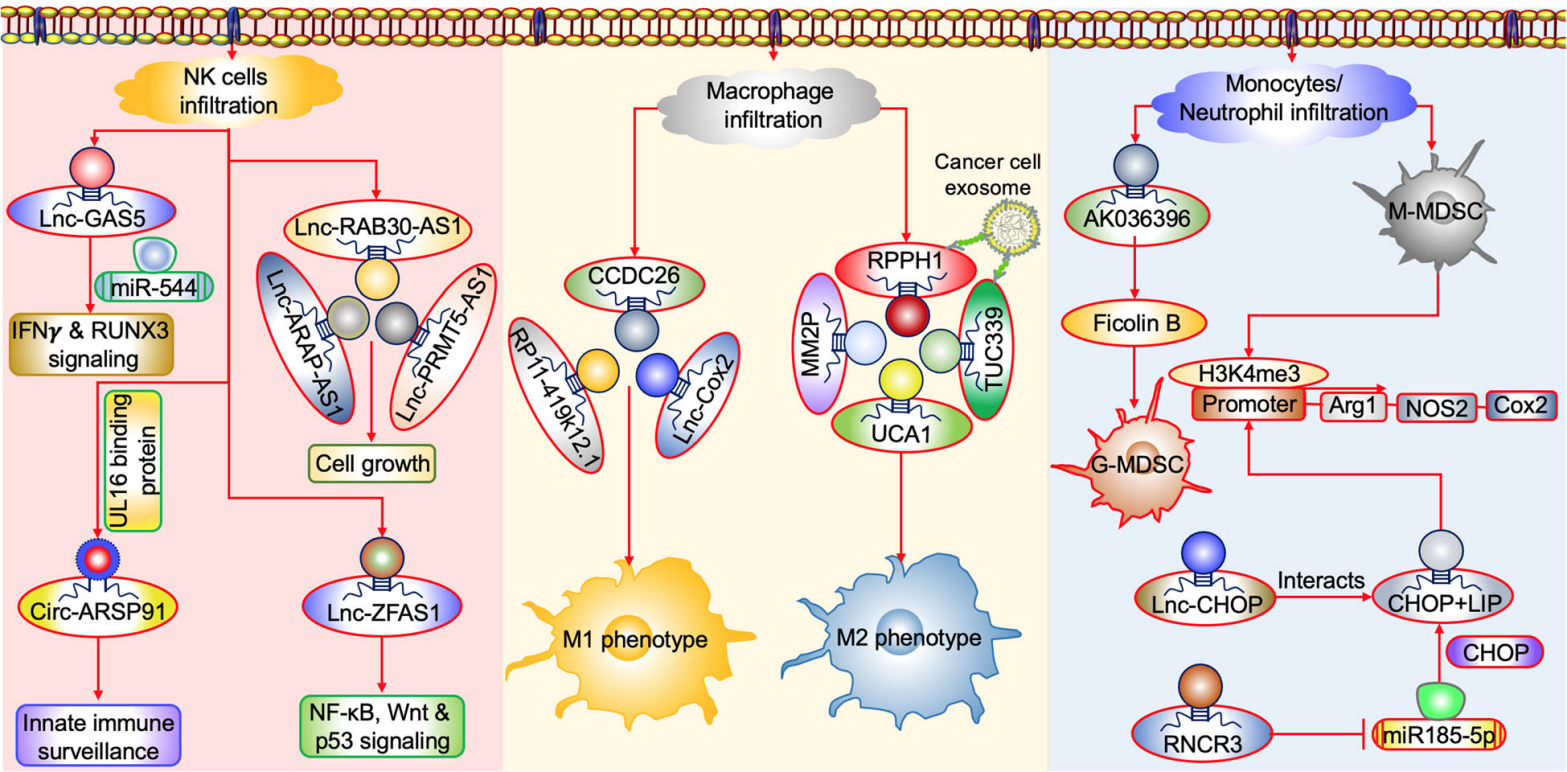
Major LncRNAs mediated molecular events occurring in the cancer immune surveillance. RPPH1 and TUC339 present in the cancer cell-secreted exosomes facilitate the expansion of M2 macrophages and MM2P, UCA1 also causes the M2 phenotype induction in cancer. On the other hand, lncRNA such as Lnc-COX2, CCDC26, and RP11-419k12.1 mediates the M1 macrophage induction. MDSC can be derived from both infiltrating neutrophils or monocytes, here AK036396 induces MDSC from infiltrating neutrophils through stabilizing the ficolin B expression. Further, Lnc-CHOP and RNCR3 both induce the H3K4 trimethylation in the promoter region of arginine, iNOS, and COX2 facilitates M-MDSC generation. NK cells infiltration consider as a potent anti-tumor response where LncGAS5 and ARSP91 facilitates the increased NK cell activity, while RAB30-AS1, ARAP-AS1, PRMT5-AS1, and ZFAS1 causes aberrant cell growth particularly in NKTCL carcinoma

Natural killer cells are important members of the innate immune system by providing a natural defence against infections involved in mediating antitumor immune responses. The growing evidence suggests the role of LncRNAs in NK cells’ development and tumor escape. A recent study [71] reported that LncRNA GAS5 is critical for IFNγ secretion, which got downregulated in NK cells of liver cancer patients. In addition, the study indicated that knocking down this GAS5 may lead to a decrease in the percentage of CD107a^+^ NKs, causing decreased apoptosis of HepG2 and Huh7 cells. However, the overexpression of LncRNA GAS5 decreased miR-544 expression, by increasing runt related transcription factor 3 (RUNX3) expression, favouring increased activated NK cell-mediated cytotoxicity suggesting a potential therapeutic target for cancer (Fig. 4).

Further, whole transcriptome analysis of natural killer/T cell lymphoma (NKTCL), normal NK and NK lymphoma cell lines revealed 166 LncRNA which had more than 1.5-fold overexpression. The LncRNAs such as RAB30-AS1, ARAP-AS1, and PRMT5-AS1 are suggested to have a biological function on cell growth [72]. In addition, Askarian-Amiri et al. (2011) [73] discovered the novel LncRNA ZNFX1 antisense RNA 1 (ZFAS1) transcribed from the antisense gene ZNFX1, which hosts for three SNORD (small nucleolar RNAs c/D box) with extreme stability for more than 16 hours. Up-regulated in cancers such as glioma, colorectal, gastric, hepatocellular, ovarian cancers and down-regulated in breast cancer. Furthermore, ZFAS1-correlated genes are associated with the upregulation of many pathways, including NF-kB signaling, b-catenin independent Wnt signaling, and p53-dependent apoptosis and the cell cycle pathways (Fig. 4) [74].

The other study revealed the role of LncRNA small nucleolar RNA host gene 12 (SNHG12) acting as a direct transcriptional target of c-Myc. Thus c-Myc-mediated increased expression of LncRNA SNHG12 facilitates proliferation and resistance to cisplatin in NKTCL [75]. This proposes SNHG12 may serve as diagnostic and druggable therapeutic target with promising clinical potential in cancer. Further, Ma et al. (2019) [76] demonstrated that circular RNA of AR-suppressed PABPC1 91 bp (ARSP91) was involved in the enhancement of cytotoxicity activity in natural killer cells against hepatocellular carcinoma. Subsequent mechanistic research revealed that upregulating UL16 binding protein 1 (ULBP1) expression in hepatocellular carcinoma cells led to the enhanced innate immune surveillance, by strengthening cytotoxic NK cells (Fig. 4).

### Decisive role of LncRNA in polarising the tissue-associated macrophages

Monocytes from the bloodstream are migrated to the tissues to differentiate as macrophages. Moreover, recent reports also evidenced that most tissue-resident macrophages originate during embryonic development [77]. They play a critical mechanism in eliminating the apoptotic bodies, harmful foreign substances, and cancer cells. Further, the macrophages can polarize to M1 that are activated macrophages involved in triggering an anti-tumor response. The M2 causes immune suppression led to the development of tumor-associated macrophages (TAMs), which play a crucial role in the tumor microenvironment as significant molecules of tumor propagation [78]. Many studies revealed the importance of LncRNA in deciding the polarization pattern of TAM.

In evidence, the LncRNA Cox2 highly expressed in M1 macrophages is induced in response to LPS and TLR1 and 2 (toll-like receptor) through MyD88 and NF-κB-dependent manner. Moreover, the silencing of this LncRNA-Cox-2 using siRNA causes a reduction in IL12 and TNF α and increased expression of M2 features with higher IL10 and arginase-1. Besides, the proliferation, migration, invasion, angiogenesis, and epithelial-mesenchymal transition (EMT) of hepatocellular carcinoma cells were increased when co-cultured with LncRNA-Cox-2 knockdown M2 macrophages, which needs to be validated in other cancers to consider as potential therapeutic target [79]. LncRNA-CASC2c expression abrogated the M2 phenotype and decreased the GBM tumor growth by inhibiting the M2 polarization [80]. Furthermore, a recent report [81] proved that the expression of LncRNA RPPH1 is associated with advanced TNM (Tumour Node metastasis) stage and owes for poor prognosis in colorectal cancer (CRC). The cancer cells release exosomes containing LncRNA RPPH1 transported into macrophages causes induced polarization of M2 phenotype. Collectively these studies indicate the necessity of targeting LncRNA, in turn, improving the immune therapeutics (Fig. 4).

Li et al. (2018) [82] showed the HCC exosome-derived Lnc-RNATUC339 is involved in the polarization of macrophages toward the M2 phenotype. In line Cao et al. (2019) [83] reported the induction of IL4 and IL13 in cell line and animal models showed upregulation of 25 LncRNA identified using microarray analysis of RAW264.7 and bone marrow-derived macrophages. Out of 25 LncRNA, the novel LncRNA MM2P is specifically expressed in M2 polarized macrophages. Further, knockdown of LncRNA MM2P blocked the M2 polarization, particularly, it weakened the angiogenesis promoting factor of M2 by inhibiting the phosphorylation of STAT6. Indicates lncRNA MM2P might involve in activation and maintenance of STAT6 causes sustained M2 polarisation. Chen et al. (2015) [84], reported that an activated LncRNA UCA1 promoted M2 macrophage infiltration, leading to tumor progression in breast cancer. In addition, the differential expression status of LncRNAs decides the fate of infiltrating CD14^+^ monocytes polarization. Gabrusiewicz et al. (2016) [85] identified the varied expression pattern of three LncRNA 14orf139, SNORA25, and CCDC26 in glioblastoma dictated the polarization of infiltrating CD14^+^TAM. Furthermore, the study highlighted that the increased expression of two LncRNAs (CCDC26 and RP11-419k12.1) favours the M1 TAM whereas the LncRNA RP11-26E5.1 was favouring M2 TAM (Fig. 4).

Chemokines play a vital role in the differentiation of macrophages in the tumor microenvironment. In specific the increased expression of CCL2 is crucial for tumor metastasis. In evidence, a recent report showed that LncRNA LNMAT1 (lymph node metastasis-associated transcript 1) recruits hnRNPL to CCL2 promotor, leads to H3K4me3 methylation causes transcriptional activation of CCL2 responsive genes. Upregulated expression of CCL2 led to the recruitment of macrophages which favours accelerated lymphatic metastasis in bladder cancer through VEGF-C secretion [86]. In line HOTAIR1 was also associated with CCL2 mediated recruitment of TAMs and MDSC [87]. LncRNA CCAT1 and NIFK-AS1 controls, the expression of miR (148a and 146a) favours the generation of M1 TAM, which was downregulated in various cancer types, causing M2 polarization and tumor cell invasion [88, 89]. Kong et al. 2018 [90] discovered a novel LncRNA-ZFPM-AS1 (ZFPM2 antisense RNA 1) was associated with gastric carcinogenesis. In addition, their results indicated the direct binding of this LncRNA to macrophage migration inhibitory factor (MIF) prevent the degradation of MIF acts as a potent destabilizer of p53. Further, knockdown of MIF caused reduced expression of ZFPM-AS1, resulting in a p53 increase in gastric cancer. Thus, the studies indicates LncRNA can function as both- oncogenes where it involved in the promotion of M2 by inhibiting the M1 polarization and tumour suppressor which facilitates the M1 generation while inhibiting the M2-related gene expression.

### Recruitment of myeloid derived suppressor cells towards tumour microenvironment through LncRNA expression

Myeloid-derived suppressor cells (MDSC) are a heterogeneous population of immature granulocytes/monocytes that accumulate in cancer, and other disease conditions like autoimmunity [91, 92]. They release arginase-1 (Arg1), NO synthase 2 (NOS2), NADPH oxidase 2 (NOX2), cyclooxygenase-2 (Cox2), and various toxic and regulatory substances like H2O2 and reactive oxygen species (ROS) [93]. Causing damage in nucleic acids, proteins, and lipids, they attenuate many immune effectors particularly curtailing the T cell activities. MDSC is classified into two major subtypes based on their cell surface phenotype and morphology: polymorphonuclear MDSC (PMN-MDSC) and monocytic MDSC (M-MDSC) [94]. MDSC is also involved in the generation of induced T regulatory cells (iTregs) [95] in tumor microenvironments causing poor survival and a low response to immune checkpoint inhibitors (ICIs). Likely, a recent study by Ma et al. (2019) [96] showed there was an increase in the MDSC counts in cancer compared to premalignant or healthy donors contributing to poor survival.

The growing evidence indicates the role of LncRNAs in recruiting these immune suppressive cells to the tumor milieu. Shang et al. (2019) [97] discovered that LncRNA Olfr29-ps1 in mouse models were 43% like the OR1F2P gene in humans. The expression of this LncRNAs is upregulated in the presence of Granulocyte-macrophage colony-stimulating factor (GM-CSF) and IL6, compared to GM-CSF, through the STAT3 and JAK3 pathways. The induced expression of this LncRNA enhances the differentiation of immune cells to M-MDSC rather than PMN-MDSC. Moreover, the N6-methyladenosine (m6A) is specific to cell type regulating the function of eukaryotic messenger RNAs (mRNA) and LncRNAs. In this context, m6A modification was increased upon proinflammatory cytokine induction. Further, this modulation favours the Olfr29-ps1 in sponging the miR-214-3p acting as a competing endogenous RNA (ceRNA) mediating in the differentiation of MDSC. Another study [98] showed the role of Lnc-CHOP mediating the differentiation of MDSC to M-MDSC through its interaction with C/EBP homologous protein (CHOP) and the C/EBPβ isoform LIP. Consequently, the interaction also promoted the epigenetic modification in H3K4me3 on the promoter region of Arg1, NOS2, NOX2, and Cox2 favours potent immunosuppressive function. Moreover, the expression of CHOP is also regulated by another LncRNA called retinal non-coding RNA3 (RNCR3) by sponging miR-185-5p [99] which affects the expansion of MDSC (Fig. 4). These reveal the complex role of LncRNA where two different non-coding RNA regulating the CHOP expression. Hence the context-dependent role of non-coding RNA activation needs to broaden might potentiates the targeted therapy in cancer.

Tian et al. 2018 [100] confirmed the role of LncRNA RUNX1 overlapping RNA (RUNXOR) in regulating the MDSC in lung cancer. The increased expression of this RUNXOR causes a decrease in the expression of runt-related transcription factor1 RUNX1 which acts as a tumour suppressor by promoting the differentiation of MDSCs in lung cancer. The expression of LncRNA was also differentially regulated in lung adenocarcinoma and squamous cell lung carcinoma indicates the screening of this LncRNA might help in identifying lung cancer subtypes. In addition, miR-9 negatively regulates the RUNX1 expression in lewis lung carcinoma. Collectively, these studies showed the negative regulation of RUNXOR and miR-9 by competing the RUNX1. Hypoxia is one of the hallmarks of the tumor microenvironment where Hypoxia-inducible factors (HIFs) play an important role in the regulation of cellular responses to hypoxia including regulation of immune cell response. Zhang et al. (2019) [101] showed the hypoxia-mediated increase in the LncRNA-plasmacytoma variant translocation 1 (Pvt1) in G-MDSC particularly by increasing the expression of c-Myc in tumour bearing mice. Moreover, the report inferred both subtypes of MDSC (M-MDSC and G-MDSC) isolated from the tumor tissue showed an upregulated expression of PVT1 with increased Arg1 and ROS expression. A recent report showed the increased expression of LncRNA AK036396 favours the immunosuppression of PMN-MDSC [102] through stabilizing the ficolin B protein that acts as a surrogate for PMN-MDSC development (Fig. 4).

### Directives of LncRNA in autophagy

Autophagy is a conserved catabolic process involved in cellular homeostasis and is required to maintain normal cellular physiology under stressful conditions [103]. It has been reported to modulate both innate and adaptive immune responses, through regulation of homeostasis, survival, activation, proliferation, and differentiation of the immune system. Moreover, it also affects the cytokine release and transport of antibodies [104, 105]. Autophagy favours immunotherapy by safeguarding the regulation of immunostimulatory signals by antigens delivery towards the immune cells, lymphocyte development, thymus selection, and immune homeostasis [106].

Several data revealed many aspects of autophagy are regulated by LncRNAs [107–109]. Specifically, one of the mechanisms by which LncRNAs regulate cellular functions is by acting as ceRNA. The MALAT1 LncRNA is a potent autophagy inducer that protects brain microvascular endothelial cells (BMEC) against oxygen-glucose deprivation/re-oxygenation (OGD/R) by sponging miR-26. In turn, it can cause upregulation of miR-26b targeting ULK2 leading to the survival of BMEC by promoting autophagy under OGD/R condition [110]. Likewise, Li et al. (2018) [111] identified the role of MALAT1 in tumorigenesis and tumor cell survival of glioma through autophagy. The study reported MALAT1 damage the suppressive effect of miR-101 on glioma cell autophagy by acting as ceRNA through upregulation of miR-101 targeting STMN1, RAB5A, and ATG4D [112].

**Fig. 5:**
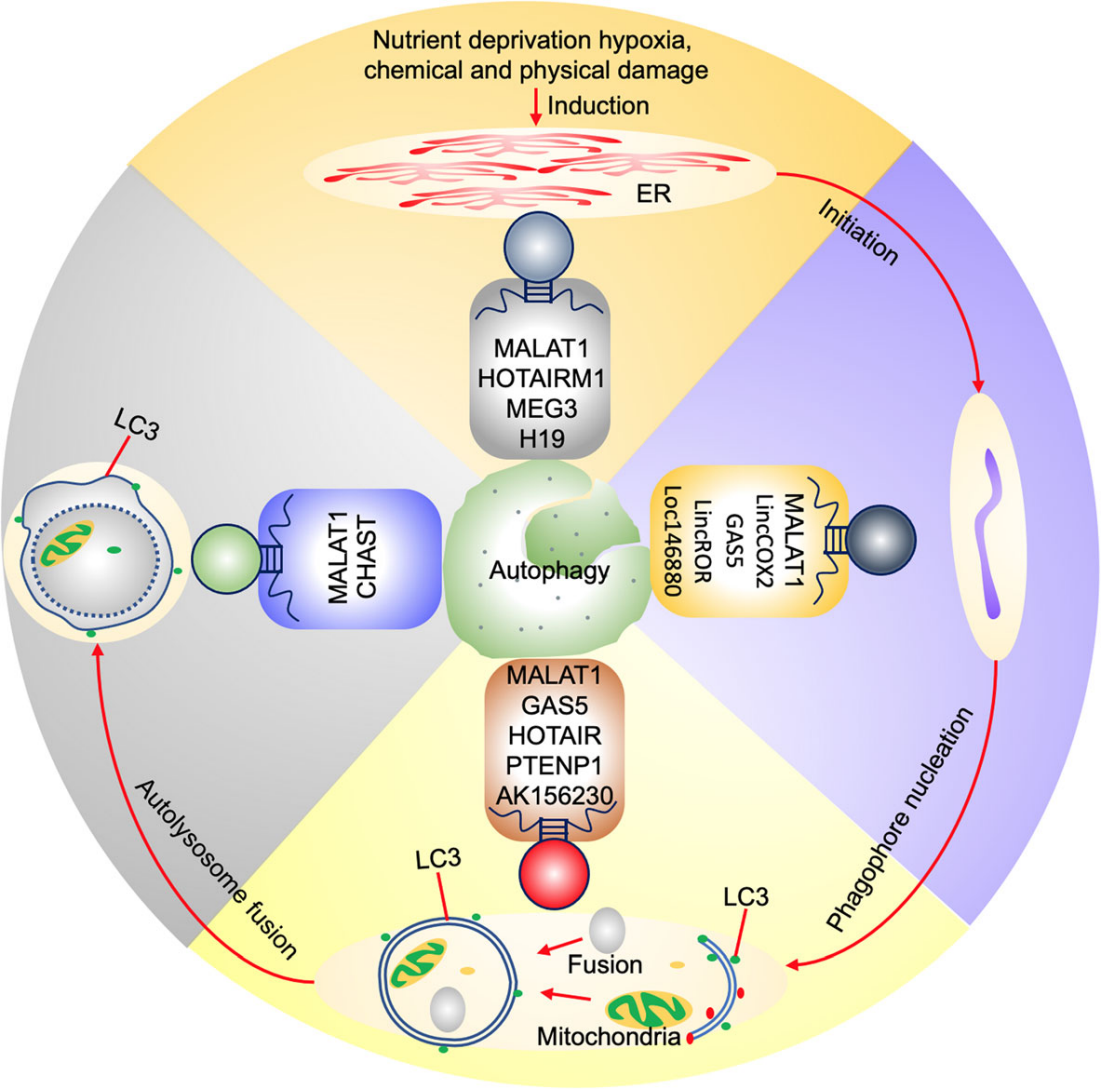
LncRNA involved in various processes of autophagy – induction, initiation, phagophore nucleation, autolysosome fusion. MALAT1 is the well-studied LncRNA involved in all the steps of autophagy. The other LncRNA GAS5 indicated its role in increased NK cell cytotoxicity also involved in the initiation and phagophore nucleation

HOTAIRM1 plays a crucial role in myeloid cell differentiation through degradation of oncoprotein PML-RARA by regulating the autophagy pathway. The study showed a decrease in the HOTAIRM1 expression in APL (acute promyelocytic leukemia) leading to the inhibition of all-trans-retinoic acid (ATRA) mediated degradation of PML-RARA causes repression of promyelocytic to granulocytic cellular differentiation. The HOTAIRM1 can regulate the autophagy pathway by acting as ceRNA by sponging the miRNAs, miR-20a/106b, and miR-125b, which targets crucial genes in autophagy through ULK1, E2F1, and DRAM2 which facilitates for the degradation of PML [113]. This reveals over-expression of HOTAIRM1 might be a potential therapeutic target for APL. The NLR family proteins NLRP3 acts as a sensor of the pathogen, environmental and host-derived factors. This NLRP3 the inflammasome is a multimeric protein complex requires for the release of proinflammatory cytokines IL-1β and IL-18 in mature form and also initiates the inflammatory form of cell death. Intriguingly, lincRNA-Cox2 could act as both an activator and suppressor of innate immune responses, playing an important role in NLRP3 inflammasome formation through binding with the NF-κB p65 subunit regulation, thus promoting its nuclear translocation and transcription. Besides, knockdown of lincRNA-Cox2 repressed inflammasome activation and prohibited the LincRNA-Cox2 triggered caspase-1 activation, causing decreased IL-1β secretion and weakened TIR-domain-containing adapter-inducing interferon-β (TRIF) cleavage, by prompting the TRIF-mediated autophagy [109] (Fig. 5).

Thus, based on the above discussion, the use of LncRNAs for cancer immunotherapy in a personalized manner can be taken as a branch of cancer therapy soon. Moreover, a recent study [114] showed the association of LncRNAs in immunotherapy responses by predictions using computational algorithms. Based on the immune-related LncRNA signature and CTL infiltration, the patients were categorized into 4 different classes. Among the four classes, the immune active class showed increased oxidative stress. Conversely, the study highlighted the patients with low LncRNA score had prolonged survival with increased immune activity compared to high LncRNA score. Furthermore, the upregulated and downregulated set of LncRNA and miRNA also act as a predictor of monitoring the chimeric antigen receptor T cells (CAR-T) therapy efficacy. Moreover, the number of LncRNAs enriched in NK cell-mediated cytotoxicity was upregulated upon CD19 CAR-T cell infusion. Particularly LncRNA RN7SL1 which was reported [114] to be associated with immune processes displayed a high degree of co-expression with histone genes including HIST1H4B and HIST1H2BL. Further, a set of 22 differentially expressed miRNA had a similar pattern in remissive patients. Especially, the expression of miR-27a-3p, a tumor suppressor in B-ALL cell lines, was upregulated after CAR-T therapy, potentially regulates a set of crosstalk genes (e.g., CEBPE and CHD4), and participates in the immune response pathways [114].

### Future prospective

The collective studies revealed the multitasking role of single LncRNA in many cancers. For instance, MALAT1 was proved to be involved in epigenetic modifications as well as autophagy induction through sponging miRNA. HOTAIR not only promotes HLAG expression [115] to enhance the antigen presentation but also down-regulates CD4^+^/CD8^+^ T-cell subsets, and also involve in autophagosome elongation and closure suggesting the effects of LncRNAs, which needs further studies to understand its mechanisms clearly. Lnc-TIM-3 and LncSNHG1 which plays a major role in the sustained expansion of exhaustive T cells might be used as the potential therapeutic target can be combined with the cell-mediated therapies like dendritic cell vaccine, adoptive T cell therapy to enhance the treatment efficacy. GAS5 which is down-regulated in many cancers likes CRC, NSCLC, breast, and pancreatic cancer indicates the over-expression of GAS5 aids in regression of the tumour. It was evidenced that LncRNA prostate cancer antigen 3 (PCA3) is overexpressed in 95% of prostate cancers and can be used as molecular markers [116] in prostate cancers, which was approved by the US food and drug administration in 2012 [117]. Furthermore, MALAT1 and CRADE can be used as a biomarker where it was detected in the serum of NSCLC and CRC in exosomal form [118]. Macrophages constitute a major population in tumour microenvironment where the increased polarization towards the M2 supports cancer proliferation. For instance, the LncRNA RPPH1 and UCA1 increase facilitates the metastasis in CRC by increasing M2 conversion needs further work in other cancer to use as a potential therapeutic target might sustain the activation of M1 macrophages. Moreover, ZFAS1 LncRNA down-regulated in breast cancer and upregulated in many cancer types indicates the context-specific regulation which needs to be studied in the future.

**Fig. 6:**
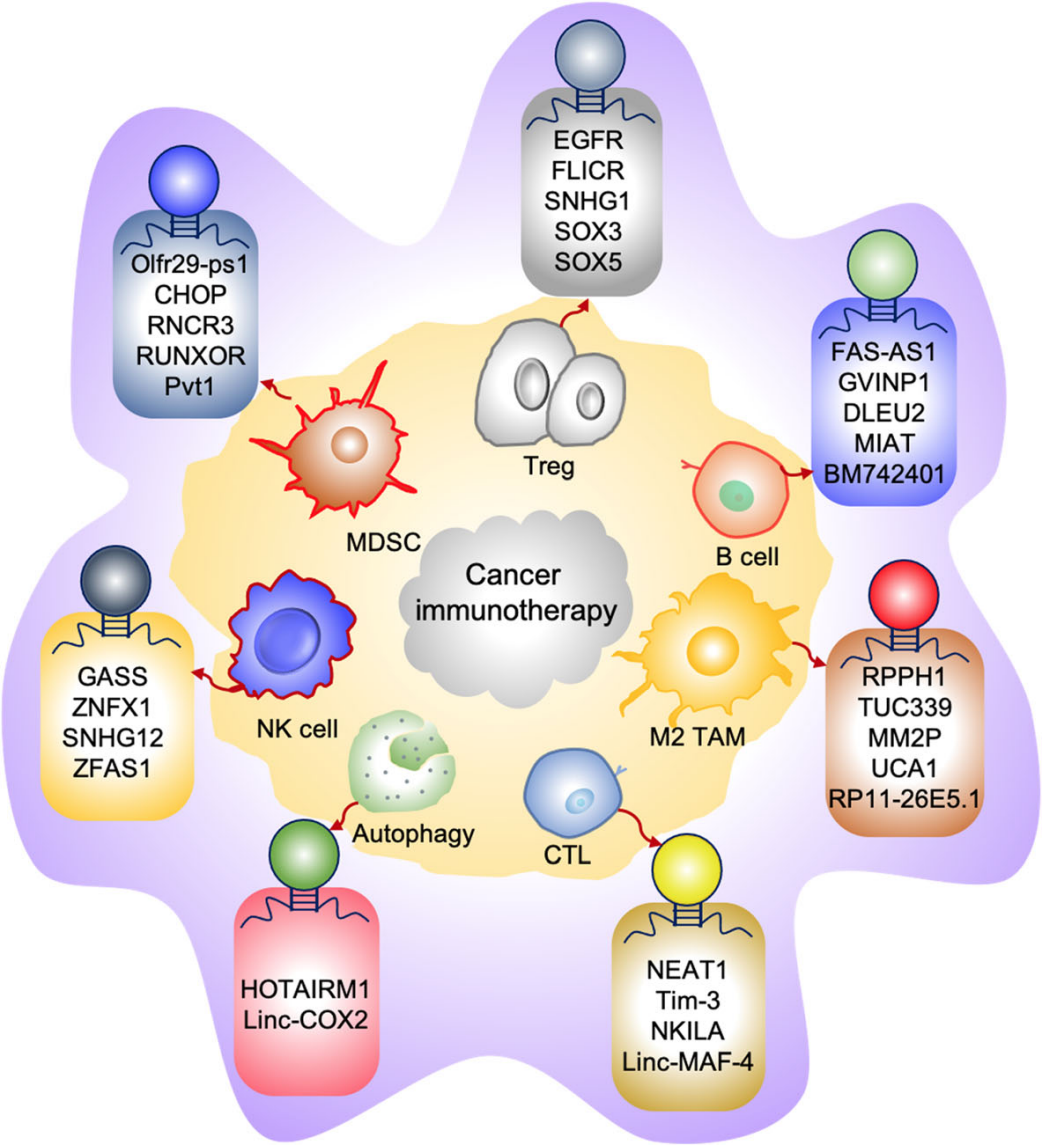
Potential LncRNAs can aid as targets for cancer immunotherapy. The figure shows relevant LncRNAs can be used as a potential target in enhancing the immunotherapeutic strategies either by upregulation of its expression (eg. GAS5 and FAS-AS1) can increase anti-tumor response or silencing of LncRNA expression (eg., MM2P and Tim-3) might mediates an increase in anti-tumor response by inhibiting the Treg, M2, MDSC expansion

Figure 6 gives an overall account of major LncRNAs which might act as a potential target in cancer immunotherapy. Moreover, Table 1 shows an account of major LncRNAs working on development of lymphoma, melanoma, and myeloma of human cancer types (courtesy to http://www.cuilab.cn/lncrnadisease) [167]. Hence, in view of the above, the LncRNA gained great potential as prognostic/diagnostic markers and therapeutic targets providing great insight into the prospects of LncRNAs in cancer immunotherapy.

**Table 1:** Account of LncRNAs responsible for blood cells related cancer and malignancies

LncRNA	Disease type	Type of interactions	Molecular functions	References
AC012065.7	Chronic lymphocytic leukemia (CLL)	Expression	Hypomethylation of AC012065.7 correlated with poor outcomes in CLL	[119]
BGLT3	Chronic myeloid leukemia (CML)	Regulation	BCR-ABL repressed LncRNA-BGL3 expression through c-Myc-dependent DNA methylation. While the ectopic expression of this lncRNA sensitize BCR-ABL transformed cell to apoptosis.	[120]
CCAT1	Acute myeloid leukemia (AML)	Expression	The over expression of CCAT1 repressed myeloid cell differentiation by sponging the tumour suppressive miR-155 leads to uncontrolled cell proliferation of human myeloid leukemia cell line HL-60.	[121]
CCDC26	AML	Regulation	CCDC26 controls growth of myeloid leukemia cells through regulation of KIT expression indicates knockdown of LncRNA expression along with ISCK03, KIT inhibitor decreased the survival of K562 cell line	[122]
p15-antisense (p15AS)	Acute lymphoblastic leukemia (ALL)	Expression	Higher expression p15AS levels facilitates the cancer progression in ALL and AML	[123]
CRNDE	CLL	Expression	CRNDE plays a major role in B cell development, found to be hypermethylation in CLL.	[119]
DLEU1	CLL, Myeloma	Mutation, Regulation, Expression, Locus mutation	DLEU2 overexpression blocks cellular proliferation and inhibits the colony-forming ability of tumor cell lines in a miR-15a/miR-16-1-dependent manner.	[124–129]
FAS-AS1	B-cell lymphoma (BCL)	NA	EZH2 is the functional component of PCR2 often over express in lymphomas, involve in hypermethylation of FAS-AS1 promoter and represses the FAS-AS1 expression leads to increased production of sFAS correlates with poor prognosis and chemoresistance.	[60]
GAS5	Liver cancer and mantle cell lymphoma	Mutation, Regulation	Down-regulation of GAS5 often detected in melanoma, BCL, and prostate, liver and breast cancer. The expression of GAS5 associated with enhanced secretion of IFNγ in NK cells and also responsible for cytotoxic and cytostatic effects of rapalogues in MCL	[71, 130–132]
BM742401	CLL	Locus	BM742401 is a tumor suppressor lncRNA inactivated by DNA methylation in CLL	[67]
HOTAIR	Acute leukemia, BCL	Regulation, Expression	HOTAIR may be regarded as a novel indicator of poor prognosis, and may serve as a potential target for gene therapy in the treatment of leukemia and lymphoma.	[133–135]
HOTAIRM1	Leukemia	Expression	HOTAIRM1 is a promising lncRNA biomarker in leukemia and solid tumors.	[136]
HOXA-AS2	NB4 promyelocytic leukemia	Regulation	ATRA induces the expression of lincHOXA-AS2 antisense non coding RNA suppress the apoptosis of cancer cell through TNF-related apoptosis-inducing ligand (TRAIL) mediated pathway.	[137]
HULC	BCL	Expression	HULC could represent a novel indicator of poor prognosis and may be served as a potential target for the diagnosis and gene therapy of diffuse large B-cell lymphoma (DLBCL).	[138]
LINC-223	Leukemia	NA	Increased expression of linc-223 arrest the cell cycle progression and contribute to monocytes differentiation in AML cells.	[139]
Lnc-NKX2-3-1 and Lnc-RTN4R1	B-cell precursor acute lymphoblastic leukemia	NA	Inhibition of Lnc-RTN4R-1 and lnc-NKX2-3-1 in ETV6/RUNX1 positive cells caused profound changes in gene expression.	[140]
LOC283177	DLBCL	NA	The lncRNA LOC283177 had a partial duplication in the C-terminal region and found to be associated with disease risk in DLBCL.	[141]
LUNAR1	T-cell acute lymphoblastic leukemia, DLBCL	Regulation	The expression of LUNAR1 is regulated by NOTCH-1, mediates the survival of T-ALL through enhancing IGF1R mRNA expression and sustains IGF1 signaling. The functionality of LUNAR1 also associates with cell proliferation and predicts a poor prognosis in DLBCL.	[142, 143]
MALAT1	Multiple myeloma (MM)	Expression	The over expression of MALAT1 was associated with increased pathogenesis of myeloma and it acts as potential indicator of early progression in cancer patients after treatment.	[69]
MEG3	Acute and chronic myeloid leukemia, Myelodysplastic syndromes (MDS) and MM	Expression, Epigenetics	The hypermethylation of MEG3 confers worst overall prognosis indicates as potential biomarker and a therapeutic target in leukemia and MM.	[144–146]
MIAT	CLL	Regulation	MIAT constitutes a regulatory loop with OCT4 in malignant mature B cell causes uncontrolled proliferation of B cells.	[66]
MINCR	Burkitt lymphoma (BL)	Regulation	MYC-induced long noncoding RNA (MINCR) had a strong positive correlation with MYC expression and identifying as a crucial player in MYC transcriptional network which able to control the cell cycle genes,	[147]
LincMONC	Myeloid leukemia	Regulation	LincRNAs MONC and MIR100HG are regulators of hematopoietic lineage decision and promotes the development of myeloid leukemia	[148]
NALT1	ALL	Regulation	Lnc-RP11-611D20.2 named NALT1 located near NOTCH1 gene regulate the NOTCH1 mediated signaling pathway through cis- regulation.	[149]
NEAT1	Leukemia	Expression	The increased expression NEAT1 facilitates multidrug resistance in leukemia by downregulating the ATP-binding cassette G2	[150]
PVT1	Acute promyelocytic leukemia, BL, Hodgkin’s lymphoma, MM, BCL	Expression, Regulation	The abnormal expression of PVT1 is correlated with uncontrolled proliferation of leukemia and lymphoma.	[151–157]
SNHG5	BCL	Locus	SNHG5 also known as U50HG member of the non-coding multiple snoRNA host gene family, is located at the breakpoint of chromosomal translocation t(3;6)(q27;q15) involved in human BCL.	[158]
T-ALL-R-LncR1	ALL	Regulation	T-ALL-R-LncR1 expressed in neoplastic T lymphocytes, knockdown of its expression associated par-4 mediated apoptosis in jurkat cell lines.	[159]
TCL6	Leukemia, Lymphoma	NA	Expressed in T-cell leukemia carrying a t(14;14)(q11;q32.1) chromosome translocation indicating a potential role in leukemogenesis.	[160]
TP73-AS1/ KIAA0495	MM	NA	Even though methylation of KIAA0495 is high in myeloma cell line, the methylation status inversely correlated with pathogenesis or progression of myeloma.	[161]
TUSC7	AML	Expression	Overexpression of the LSAMP and TUSC7 genes in acute myeloid leukemia associated with position effect in an AML patient bearing a 3q13.31 microdeletion	[162]
UCA1	AML	Regulation	UCA1 maintains proliferation of AML cells by inhibiting p27kip1cell cycle regulator.	[163]
WTI-AS	AML	Epigenetics, Interaction	WT-1 is a developmental gene was found mutated in Wilms tumour and AML encodes the WT1-AS acts as important regulator of WT-1 expression.	[164, 165]
XIST	B-cell acute lymphoblastic leukemia	NA	The expression of XIST plays a crucial role in growth of B-ALL might serve as potential therapeutic target.	[166]

Expression: Expression refers to the upregulation of the effector genes; Regulation: Regulation refers to the cis/trans-regulation of the effector genes; Epigenetic interaction: Interactions resulting in the epigenetic effects; Mutation: Interaction causing mutation of the gene causing effects in targets other than same locus; Locus mutation: Interaction causing mutation of the gene causing effects in same locus.

## Conclusion

The functionality of LncRNA is complex and competes with both miRNA and mRNA to regulate the expression of the protein. Recent studies revealed that LncRNAs act as epigenetic modifiers involved in promoting innate immune memory responses. Future explorations of immune-related LncRNAs in predicting immune responses are needed, as potential candidatures for specific immune cell-mediated tumor evasion and cellular apoptosis.

## Data Availability

Not applicable.

## Abbreviations

AICD: Activation induced cell death
ALL: Acute lymphoblastic leukemia
AML: Acute myeloid leukemia
APL: Acute promyelocytic leukemia
Arg1: Arginase-1
ARSP91: AR-suppressed PABPC1 91 bp
ATRA: All-trans-retinoic acid
BCL: B-cell lymphoma
BIC: B-cell integration cluster
BM: Burkitt lymphoma
BMEC: Brain microvascular endothelial cells
CAR-T: Chimeric antigen receptor T cells
CECR7: Cat eye syndrome chromosome region, candidate 7
ceRNA: Competing endogenous RNA
CHOP: C/EBP homologous protein
CI: Cancer immunotherapy
CLL: Chronic lymphocytic leukemia
CML: Chronic myeloid leukemia
CNS-2: Conserved noncoding sequence-2
Cox2: Cyclooxygenase-2
CRNDE: Colorectal neoplasia differentially expressed
CRC: Colorectal cancer
CTLs: Cytotoxic T lymphocytes
DAMPs: Damage-associated molecular patterns
DCs: Dendritic cells
DLBCL: Diffuse large B-cell lymphoma
EGFR: Epidermal growth factor receptor
EMT: Epithelial-mesenchymal transition
EZH2: Enhance of Zeste Homolog 2
Flicr: Foxp3 long intergenic noncoding RNA
GBM: Glioblastoma multiforme
GM-CSF: Granulocyte-macrophage colony-stimulating factor
G-MDSC: Granulocytes-MDSC
HDAC: Histone-deacetylase
HIFs: Hypoxia-inducible factors
ICIs: Immune checkpoint inhibitors
IFN γ: Interferon γ
iTregs: induced T regulatory cells
LincRNA: Long intergenic non-coding RNA
LncRNA NKILA: Nuclear factor κB interacting long non coding RNA
LncRNAs: Long non-coding RNAs
LNMAT1: Lymph node metastasis-associated transcript 1
LSD1: Lysine specific demethylase 1
M-MDSC: Monocytic MDSC
MDS: Myelodysplastic syndromes
MDSC: Myeloid-derived suppressor cells
MIF: Macrophage migration inhibitory factor
MM: Multiple myeloma
mRNA: messenger RNA
nNEAT1: Nuclear-enriched autosomal transcript 1
NeST: Nettoie Salmonella pas Theiler’s
NKs: Natural killer cells
NKTCL: Natural killer/T cell lymphoma
NOS2: NO synthase 2
NOX2: NADPH oxidase 2
OGD/R: Oxygen-glucose deprivation/re-oxygenation
p-ATM: PD-L1, ataxia telangiectasia mutated kinase
PAMPs: Pathogen-associated molecular patterns
PMN-MDSC: Polymorphonuclear MDSC
PRC: Polycomb group protein
Pvt1: Plasmacytoma variant translocation 1
RBM5: RNA binding motif protein5
RNCR3: Retinal non-coding RNA3
ROS: Reactive oxygen species
RUNX: Runt related transcription factor
RUNXOR: RUNX1 overlapping RNA
sFAS: soluble FAS receptor
SNHG1: Small nucleolar RNA host gene 1
SNHG20: Small nucleolar RNA host gene 20
SNORD: Small nucleolar RNAs C/D box
TAMs: Tumor-associated macrophages
TCR: T cell receptor
TILs: Tumor-infiltrating lymphocytes
TLR: Toll-like receptor
TNM: Tumour node metastasis
TNF: Tumour necrosis factor
TRAIL: TNF-related apoptosis-inducing ligand
Tregs: T regulatory cells
TRIF: TIR-domain-containing adapter-inducing interferon-β
ULBP1: UL16 binding protein 1
ZFAS1: ZNFX1 antisense RNA 1
ZFPM-AS1: ZFPM2 antisense RNA 1
